# Engineering Leaf-Like UiO-66-SO_3_H Membranes for Selective Transport of Cations

**DOI:** 10.1007/s40820-020-0386-6

**Published:** 2020-02-17

**Authors:** Tingting Xu, Muhammad Aamir Shehzad, Xin Wang, Bin Wu, Liang Ge, Tongwen Xu

**Affiliations:** 1 CAS Key Laboratory of Soft Matter Chemistry, iCHEM (Collaborative Innovation Center of Chemistry for Energy Materials), Department of Applied Chemistry, School of Chemistry and Materials Science, University of Science and Technology of China, Hefei, 230026 People’s Republic of China; 2 School of Chemistry and Chemical Engineering, Key Laboratory of Environment-Friendly Polymeric Materials of Anhui Province, Anhui University, Hefei, 230601 People’s Republic of China

**Keywords:** Metal–organic frameworks, In situ smart growth, UiO-66-SO_3_H membrane, Ion separation

## Abstract

Metal–organic frameworks (MOFs) with angstrom-sized pores are promising functional nanomaterials for the fabrication of cation permselective membranes (MOF-CPMs). However, only a few research reports show successful preparation of the MOF-CPMs with good cation separation performance due to several inherent problems in MOFs, such as arduous self-assembly, poor water resistance, and tedious fabrication strategies. Besides, low cation permeation flux due to the absence of the cation permeation assisting functionalities in MOFs is another big issue, which limits their widespread use in membrane technology. Therefore, it is necessary to fabricate functional MOF-CPMs using simplistic strategies to improve cation permeation. In this context, we report a facile in situ smart growth strategy to successfully produce ultrathin (< 600 nm) and leaf-like UiO-66-SO_3_H membranes at the surface of anodic alumina oxide. The physicochemical characterizations confirm that sulfonated angstrom-sized ion transport channels exist in the as-prepared UiO-66-SO_3_H membranes, which accelerate the cation permeation (~ 3× faster than non-functionalized UiO-66 membrane) and achieve a high ion selectivity (Na^+^/Mg^2+^ > 140). The outstanding cation separation performance validates the importance of introducing sulfonic acid groups in MOF-CPMs.

**Electronic supplementary material:**

The online version of this article (10.1007/s40820-020-0386-6) contains supplementary material, which is available to authorized users.

## Highlights

- Ultrathin (< 600 nm) and defect-free leaf-like UiO-66-SO_3_H membranes were fabricated via in situ smart growth.
- The sulfonated angstrom-sized ion transport channels in the membranes could accelerate the cation permeation (~ 3×  faster than non-functionalized UiO-66 membrane) and achieve a high ion selectivity (Na^+^/Mg^2+^ > 140).

**Electronic supplementary material:**

The online version of this article (10.1007/s40820-020-0386-6) contains supplementary material, which is available to authorized users.

## Graphical abstract

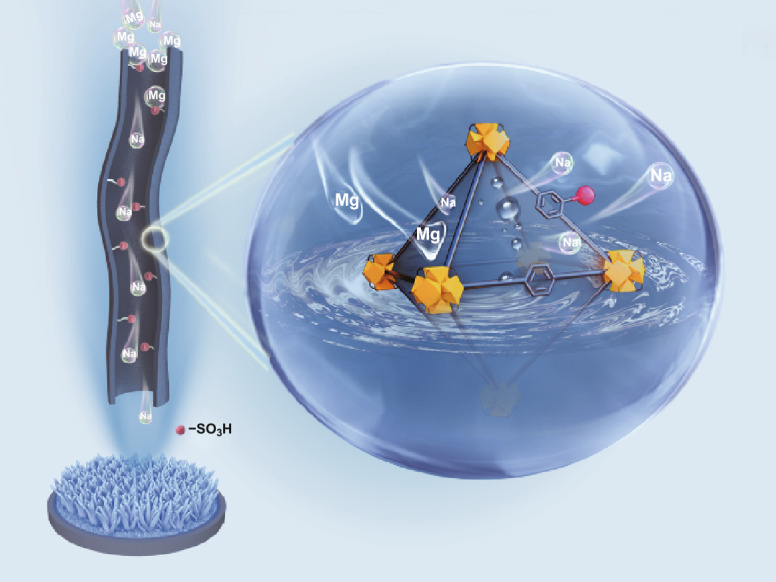

## Introduction

Metal–organic frameworks (MOFs) are famous potential candidates in membrane-integrated separation processes due to their angstrom-sized pores [1–3]. Previously, MOF membranes have shown successful use in gas separation [4, 5], pervaporation [6, 7], organic solvent nanofiltration [8], and dye separation [9, 10]. However, effectual use of the MOFs in extracting valuable metal cations from salt lakes and seawater is still a problem, which urges membrane scientists to find a proper answer. The inherent sub-nanometer pores in the MOFs matching with the sizes of valuable metal cations [11, 12] are motivational for selective transport and separation of ions [13–15].

Nonetheless, difficult self-assembling and poor water resistance still perturb their effectual use in membranes and only a few studies show the fabrication of defect-free membranes of ZIF-8 [16, 17], MIL-53 [18], and zirconium (IV)-based MOFs (such as UiO-66-NH_2_) [19, 20]. The successfully fabricated MOF membranes have certainly exhibited promising desalination performance. However, we have only observed limited use of MOF membranes for selective transport and separation of cations [12–14]. Guo et al. [12] constructed polystyrene sulfonate threaded HKUST-1 (PSS@HKUST-1) membranes on anodic alumina oxide (AAO) substrates to achieve high lithium ion selectivity. The results convinced that MOFs as a very promising material can be used for the efficient separation of cation ions. Most recently, Zhang et al. [13] have proposed a ZIF-8/GO composite membrane for selective separation of alkali metal ions. The membrane exhibited a high LiCl/RbCl selectivity of ~ 4.6, which is much greater than the measured selectivity of traditional porous membranes (0.6–0.8). Our recent work also complemented the use of MOF membrane for ion separation. The fabricated UiO-66-NH_2_ membranes exhibited ultrahigh ion selectivity performance (Na^+^/Mg^2+^ > 200 and Li^+^/Mg^2+^ > 60) [14]. However, the cation permeation of the membranes is not good as its excellent selectivity, even though the MOF layer is ultrathin. The slow cation permeation is due to the absence of the permeation assisting groups (e.g., acetate or sulfonate) in the MOFs, which is a big issue and limits the widespread energy-efficient use of the MOF-CPMs in the membrane technology applications. Thus, the technological inventions and chemical functionalization are both highly desirable for fabrication of the defect-free MOF-CPMs with fast cation permeation and selectivity.

**Scheme 1:**
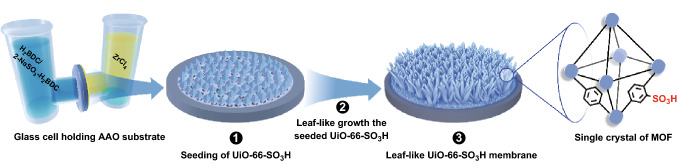
A simplistic three-step route to in situ grow the leaf-like membranes. Step 1: seeding of the UiO-66-SO_3_H at the surface of AAO substrate. Step 2: the in situ smart growth of leaf-like UiO-66-SO_3_H nanostructures in such a way. Step 3: fabrication of the highly decorated UiO-66-SO_3_H membrane at the AAO surface

In this context, we hereby report the fabrication of defect-free functionalized MOF-CPMs to improve cation permeation and achieve high ion selectivity. We propose in situ smart growth of the leaf-like UiO-66-SO_3_H membranes within a self-designed two-compartment reaction cell (Scheme 1). Herein, the solutions containing metal ions and ligands are separated by an AAO substrate. Both the solutions diffuse toward opposite direction by passing through the pores of the AAO substrate and meet at the AAO surface to crystallize UiO-66-SO_3_H. The nucleation initiates from the seeds and in situ produces ultrathin leaf-like UiO-66-SO_3_H membranes (< 600 nm). The sulfur content in the UiO-66-SO_3_H layer is easily controlled by tuning the feed ratio of the ligands. Thus, the produced leaf-like UiO-66-SO_3_H membranes benefiting from the sulfonated angstrom-sized ion transport channels are in anticipation of accelerating cation permeation and achieving cation selectivity.

## Experimental Section

### Materials

Zirconium (IV) chloride (ZrCl_4_, 98%) was purchased from Shanghai Macklin Biochemical Co., Ltd. (Shanghai, China). Terephthalic acid (H_2_BDC, 98%) and monosodium 2-sulfoterephthalate (2-NaSO_3_–H_2_BDC, 98%) were purchased from TCI (Shanghai, China) development Co., Ltd. *N*,*N*-dimethylformamide (DMF), hydrochloric acid (HCl, 37%), acetic acid (CH_3_COOH, 99.5%), KCl, NaCl, LiCl, and MgCl_2_ were of analytical grade and obtained from China National Pharmaceutical Group Industry Co., Ltd. (Beijing, China). All reagents and solvents were commercially available and used as received. Deionized water was used throughout the experiments.

Anion exchange membrane Neosepta AMX (Tokuyama Co., Japan) was used in electrodialysis (ED) experiments. Anodic alumina oxide (AAO) substrates were obtained from Hefei Pu-Yuan Nano Technology Ltd.

### Preparation of UiO-66 Nanoparticles

ZrCl_4_ (0.466 g) and H_2_BDC (0.332 g) were first dissolved in 64 mL DMF; then, 8 mL of concentrated HCl and 8 mL of CH_3_COOH were added. The mixture was sonicated for 20 min until fully dissolved. After that, the vessel was heated at 80 °C for 24 h in the oven. Then, the UiO-66 nanoparticles were collected by centrifugation and then washed with DMF and ethanol, respectively, for at least three times. At last, the nanoparticles were dried at 80 °C for 12 h.

### Preparation of UiO-66-SO_3_H Nanoparticles

**Fig. 1:**
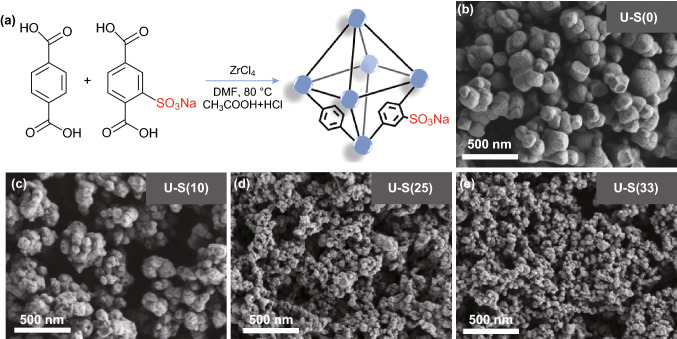
**a** Route toward the synthesis of UiO-66-SO_3_H nanoparticles. SEM micrographs of **b** U-S(0), **c** U-S(10), **d** U-S(25), and **e** U-S(33) nanoparticles

To obtain the UiO-66-SO_3_H with different sulfonic acid group contents, the procedure followed is the same as the synthesis of UiO-66 nanoparticles, except for the addition of nominal stoichiometric amounts of H_2_BDC and 2-NaSO_3_–H_2_BDC, as shown in Fig. 1a. UiO-66-SO_3_H with different sulfur contents are denoted as U-S(X), in which X represents the percentage of 2-NaSO_3_–H_2_BDC to H_2_BDC, where *X *= 0, 10, 25, 33 (*X *= 0, U-S(0) represent for UiO-66). For example, when *X *= 25, 0.249 g of H_2_BDC and 0.134 g of 2-NaSO_3_–H_2_BDC were used. Then, repeat the same wash operation as preparation of UiO-66 to obtain the dried UiO-66-SO_3_H nanoparticles.

### Preparation of UiO-66-SO_3_H Membranes

AAO substrate with a pore size of 55 ± 15 nm and Ø 25 mm diameter was mounted on a self-designed two-compartment reaction cell, where the H_2_BDC/2-NaSO_3_–H_2_BDC solution and ZrCl_4_ solution were separated by the AAO substrate.

The H_2_BDC solution was prepared by dissolving 0.332 g H_2_BDC in 32 mL of DMF, then 4 mL concentrated HCl and 4 mL CH_3_COOH were added, and the mixture was sonicated for 20 min until fully dissolved. Meanwhile, the ZrCl_4_ solution was prepared by dissolving 0.466 g of ZrCl_4_ in 40 mL of DMF and then sonicated for 20 min until fully dissolved. The ligand and the metal salt solution were then added in different reaction cells before being heated at 80 °C for 24 h. Then, the MOF membranes were taken out and washed with DMF and stored in ethanol before use.

Here, we prepared membranes with percentages of 2-NaSO_3_–H_2_BDC to H_2_BDC being 0%, 10%, 25%, and 33%, denoted as U-SM(0), U-SM(10), U-SM(25), and U-SM(33) (Table S1).

### Characterizations of UiO-66-SO_3_H Nanoparticles

The morphology of the nanoparticles was characterized using a field emission scanning electron microscope (FESEM, Gemini 500, Germany). The chemical composition of the nanoparticles was investigated using Fourier transform infrared spectrometer (FTIR, Thermo Nicolet FTIR spectrometer, USA). Powder X-ray diffraction (PXRD) patterns were obtained using a Rigaku X-ray Diffractometer Model TTR-III (Tokyo, Japan). TGA thermograms were recorded using a TGA Q5000 V3.15 analyzer at a heating rate of 10 °C min^−1^ under N_2_ atmosphere. Brunauer–Emmett–Teller (BET) gas sorptometry measurements were performed on an Autosorb iQ (Quantachrome, Autosorb iQ, USA) at 77 K. Before each isotherm, approximately 200 mg of activated MOF samples was activated by heating for 5 h under high vacuum at 120 °C. Element analysis (EA) was tested by Elementar (Vario EL/micro cube, Germany).

### Membrane Characterization and Performance Assessment

#### Structure Characterizations

The morphology of the surface and cross section was characterized using a field emission scanning electron microscope (FESEM, Gemini 500, Germany). Grazing incidence X-ray diffraction (GIXRD) was measured by X-ray diffraction spectrometer (PANalytical X’pert PRO MPD, Holland).

#### Permselectivity Measurements

Electrodialysis (ED) performance was evaluated referring to our previous work [21]. The configuration of ED device comprises four compartments, including anode, diluted, concentrated, and cathode compartment. Here, ED experiments were performed at a current density of 5 mA cm^−2^ and the effective area of the membrane was 2 cm^2^. Note that the MOF layer was faced to the diluted compartment. Briefly, for binary solution separation, a 100 mL Na_2_SO_4_ solution (0.3 mol L^−1^) is used for anode and cathode compartments, 100 mL 0.1 mol L^−1^ NaCl and 0.1 mol L^−1^ MgCl_2_ (or 0.1 mol L^−1^ KCl and 0.1 mol L^−1^ MgCl_2_/0.1 mol L^−1^ LiCl and 0.1 mol L^−1^ MgCl_2_) mixture solution for diluted compartment, and 100 mL 0.01 mol L^−1^ KCl solution for concentrated compartment. For single-component solution, the diluted compartment employed 100 mL 0.1 mol L^−1^ KCl (or NaCl, LiCl, and MgCl_2_); other compartments followed the separation condition of binary solutions. All solutions were circulated by peristaltic pumps, and each experiment lasted for 1 h. After one test finished, the samples were drawn from concentrated compartment and the testing apparatus was thoroughly washed with DI water for 30 min. The concentration of ions was measured by inductively coupled plasma optical emission spectrometer (ICP-OES, Optima 7300DV, USA). The cation permeation through the test membranes was calculated by the change in the concentrated compartment compared with the diluted compartment. Herein, the cation permselectivity of the membranes was simply calculated as the ratio of monovalent ion and divalent ion permeation fluxes. The permeation and permselectivity in this work were calculated referring to our previous work [14].

## Results and Discussion

### Characterization of U-S(X) Nanoparticles

**Fig. 2:**
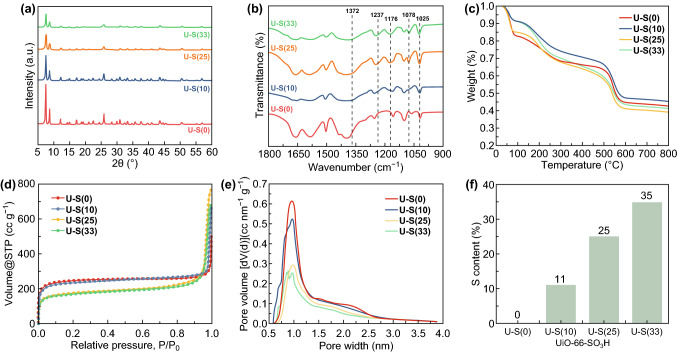
**a** XRD patterns, **b** FTIR, **c** TG, **d** N_2_ adsorption measurement, **e** pore size distribution, and **f** sulfur content of U-S(X) nanoparticles

The addition of modulators plays an important role in the synthesis of UiO-66-SO_3_H [22–24]. Based on our preliminary experimental investigations (Figs. S1-S3 and Figs. 1, 2) to grow UiO-66-SO_3_H (take U-S(25) as an example) without using any acid, using a single acid (CH_3_COOH or HCl), and both the acids (CH_3_COOH and HCl), we observed that the option of using both the acids is best suited to acquire the required UiO-66-SO_3_H (Figs. 1 and 2). SEM images of U-S(X) nanoparticles are shown in Fig. 1b–e. One can see that the size of U-S(X) nanoparticles gradually decreases with the increasing 2-NaSO_3_–H_2_BDC content due to the competition between organic linkers. In other words, significant deterioration of MOF structure will happen at higher 2-NaSO_3_–H_2_BDC content [22].

The structures of U-S(X) nanoparticles were confirmed by PXRD, FTIR, TG analyses, N_2_ adsorption, and element analysis. The crystallinity of U-S(X) was examined by PXRD (Fig. 2a), which gave sharp diffraction lines coincided with that of pristine UiO-66 [25]. The results clearly indicate that the introduction of sulfonic acid groups did not change the crystal structure of UiO-66. The XRD intensity apparently decreases with increasing the sulfonic acid ligand, which means deterioration of MOF structure gradually happened with increasing the degree of 2-NaSO_3_–H_2_BDC content [22]. These results are consistent with SEM results; the lower intensity means lower crystallinity and smaller particle. The presence of sulfonic acid groups of U-S(X) nanoparticles was confirmed by FTIR spectroscopy (Fig. 2b). The absorption bands at 1237 and 1176 cm^−1^ and the shoulder peaks at around 1372 cm^−1^ are assigned to the symmetric and asymmetric stretching modes of O=S=O bonding, respectively [26]. The band appeared at 1078 cm^−1^ corresponds to the *n*-plane skeletal vibration of the benzene rings substituted by a sulfonic acid group [27]. The band at 1025 cm^−1^ is assigned to the stretching mode of S=O bonding. Because of the presence of the aromatic ring, there is a slight shift from its original position at 1030 cm^−1^ [22, 26, 28]. The intensity of these bands increased with increasing the fraction of sulfonated ligand. Furthermore, the stability of U-S(X) nanoparticles was examined by immersing the as-prepared samples in mixed saline solutions (0.1 mol L^−1^ NaCl/0.1 mol L^−1^ MgCl_2_). As shown in Fig. S4, XRD and FTIR results of U-S(X) exhibit excellent stability in various saline solutions after 120 h, which provides a guarantee for the electrodialysis test. In TG profiles (Fig. 2c), all samples show very similar weight losses, indicating that the addition of 2-NaSO_3_–H_2_BDC ligand has little effect on the thermal stability of the UiO-66 framework. The weight loss in range of 400–550 °C is attributed to structure collapse and loss of organic linkers [22].

The N_2_ adsorption measurement was applied to characterize the textural properties of the fabricated nanoparticles (Fig. 2d). The amount of adsorbed N_2_ decreases with the increasing content of sulfonic acid ligand. The MOF cavity size is decreased after introducing—SO_3_H groups, which can also be evidenced by the decrease in pore volume (Fig. 2e). If we continuously increase the 2-NaSO_3_–H_2_BDC content, the adsorption would decrease to zero finally [23, 24]. The BET surface area values of the U-S(X) nanoparticles were calculated and are listed in Table S1. The pore size distribution calculated from the isotherm using the SF model shows that most of the pores of all U-S(X) fall into the size range of 7 to 11 Å (Fig. 2e). However, distribution of pore size widens with the increase in sulfonic acid group content, which would affect the separation performance. Element analyses were tested to make sure the content of sulfonic acid linker in U-S(X). As shown in Fig. 2f, the resulting contents of sulfonic acid linker in U-S(X) are almost consistent with the feed ratio.

### Characterization of UiO-66-SO_3_H Membranes

**Fig. 3:**
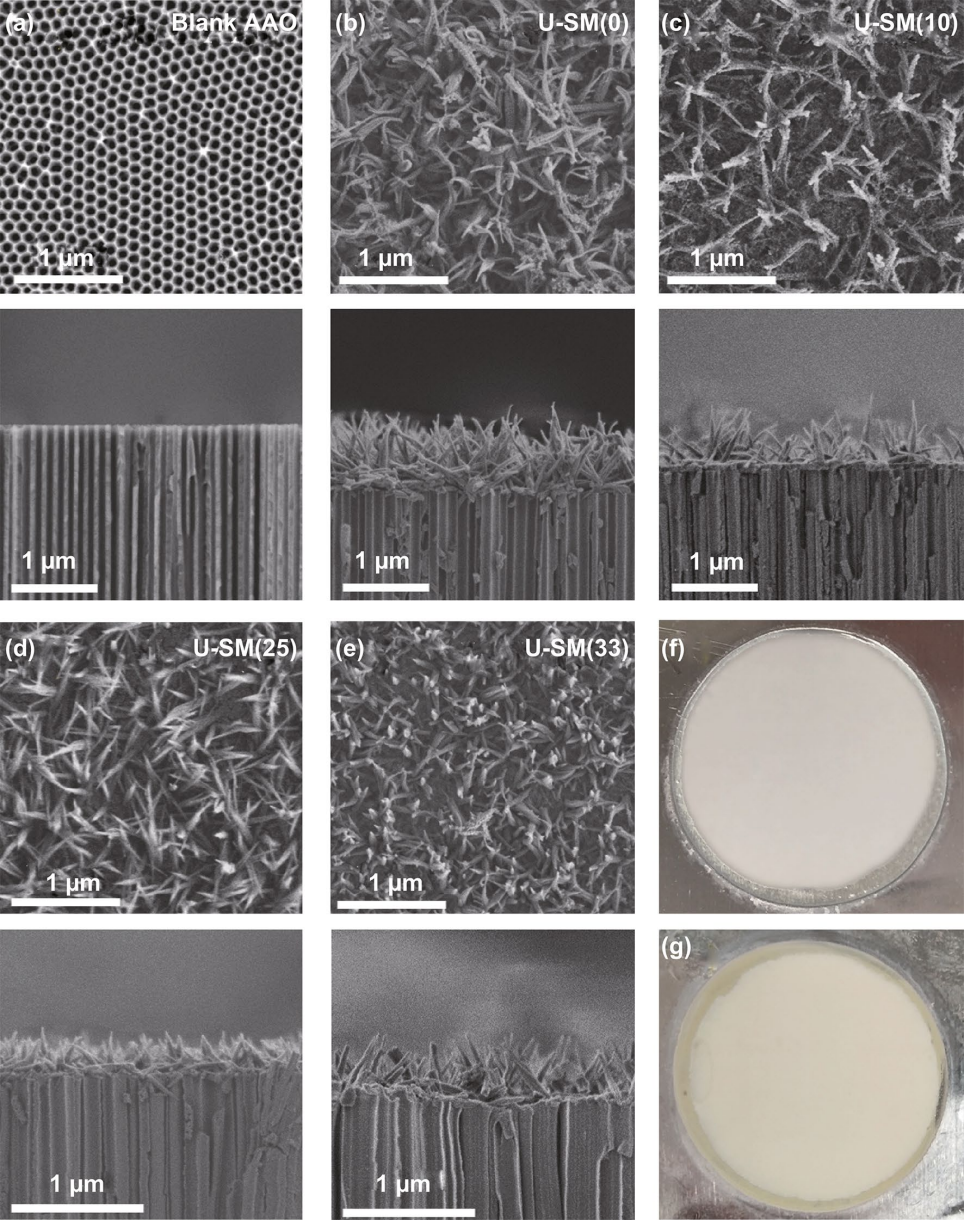
SEM surface and cross-sectional images of **a** blank AAO, **b** U-SM(0), **c** U-SM(10), **d** U-SM(25), and **e** U-SM(33). Photographs of the **f** blank AAO and **g** representative U-SM(25)

The successful deposition of a dense U-S(X) crystalline layer on the AAO was achieved by optimizing the preparation parameters and conditions. The optimized recipe is shown in Table S2. The resulting U-SM(X)s were characterized by SEM (Fig. 3b–e; Fig. 3a shows the images of blank AAO). A continuous polycrystalline U-S(X) layer is formed on the AAO substrate without any visible cracks (Fig. S5). For all the U-SM(X)s, the surfaces facing to ligand solution side were the highly decorated with leaf-like U-S(X) layer; at the salt solution side, U-S(X) nanoparticles grew scatteredly on the AAO surface. The morphologies of U-SM(X)s facing mixed ligand side (Figs. 3 and S6) should arise from different local molar ratios of ligand/salt [29–31]. On the ligand side, the local ligand–salt molar ratio should be far greater than the designed molar ratio, and the presence of hydrochloric acid speeds up formation for the case of U-S(X) nanocrystals, which are produced to form continuous membrane [32, 33]. The seeding of U-S(X) integrated with the substrate closely because the coordination bonds between the carboxylate oxygens and aluminum atoms from substrate were constructed, which favored the nucleation and growth of U-S(X) on the alumina substrate [14, 20, 34]. The U-S(X) layers are 300–800 nm in thickness, substantially thinner than most of the UiO-type membranes reported so far [6, 7, 20, 35]. However, decoration level (density of the grown U-S(X) leaves) of U-SM(X)s varies with the addition of sulfonic acid group; surface coverage of “leaves” becomes sparse. This should be the competition between mixed ligands (H_2_BDC and 2-NaSO_3_–H_2_BDC) that makes the growth environment not so stable. Figure 3f, g shows the photographs of blank AAO and representative U-SM(25), respectively.

**Fig. 4:**
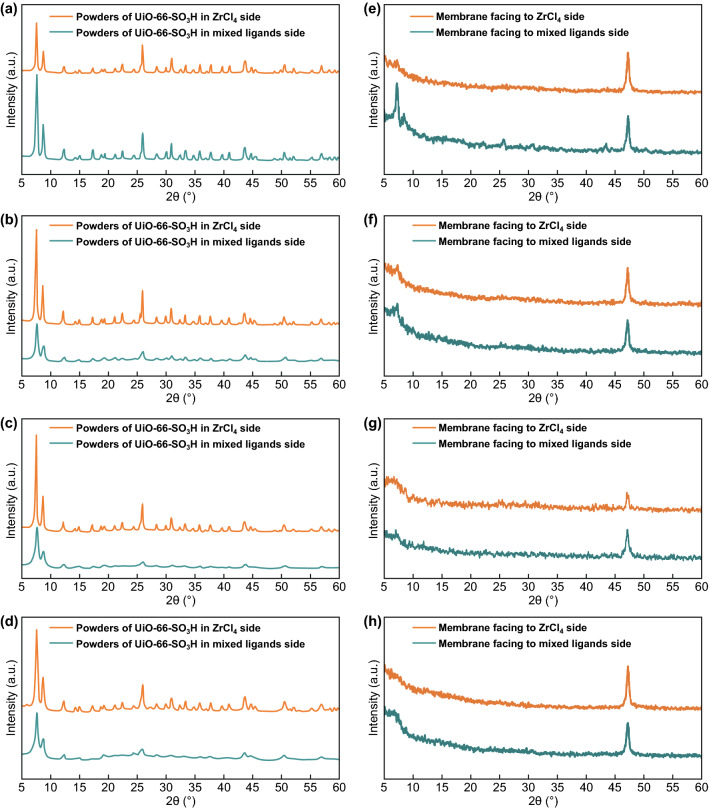
PXRD patterns of nanoparticles from two chambers in the preparation process of **a** U-SM(0), **b** U-SM(10), **c** U-SM(25), and **d** U-SM(33). GIXRD patterns of **e** U-SM(0), **f** U-SM(10), **g** U-SM(25), and **h** U-SM(33)

As the diffusion rate is very fast at the beginning, the reaction solution diffuses to the other chamber and forms U-S(X) crystals in the chambers. The generated U-S(X) nanoparticles in both chambers are proved by PXRD (Fig. 4a–d). Because the UiO-66-SO_3_H leaves on the membrane surface are sparse, the actual effective thickness of the membrane is smaller than the length of the leaves. Here, GIXRD was employed (Fig. 4e–h) to test the U-SM(X)s. From the GIXRD patterns, diffraction lines were not sharp because the top of U-SM(X)s’ layers was sparse and the membrane is too thin.

### High Cation Permselective Separation Performance of U-SM(X) Membranes

**Fig. 5:**
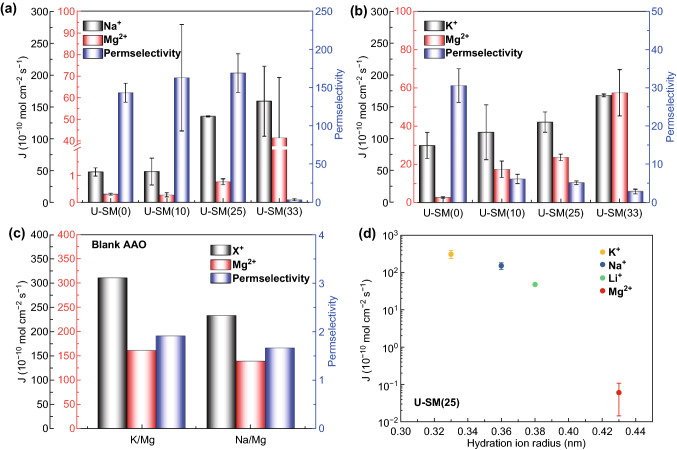
**a** Na^+^/Mg^2+^ and **b** K^+^/Mg^2+^ separation performance of U-SM(X)s, **c** K^+^/Mg^2+^ and Na^+^/Mg^2+^ separation performance of blank AAO, **d** permeation of 0.1 mol L^−1^ NaCl/KCl//LiCl/MgCl_2_ solution of U-SM(25)

The cation selective separation performance for all the U-SM(X)s was investigated using a laboratory-scale electrodialysis stack [36]. As shown in Fig. 5a, all the U-SM(X)s exhibit cation permeation in the order of Na^+^ > Mg^2+^, which is based on their hydrated diameters (Na^+^: 7.2 Å, Mg^2+^: 8.6 Å [11]). Specifically, U-SM(25) exhibits a three-times ion permeation compared with U-SM(0) but with a tiny change of selectivity. The sulfonic acid groups in ion channels evidently accelerate the transfer of Na^+^. Meanwhile, the MOF nanostructure still holds the appropriate pore size for Na^+^ selective transport to ensure the high selectivity. However, ion selective performance of U-SM(33) reduces quickly because the permeation of Mg^2+^ increases a lot. This might be due to the pore size distribution that widens with the increased content of sulfonic acid groups of UiO-66-SO_3_H, which certainly weakens the size sieving effect. Herein, the binding affinity of sulfonate groups to cations is also a possible reason behind the improved cation permeation and reduced ion selectivity [37, 38]. Briefly, the binding affinity of –SO_3_H groups to the divalent cations compared with the monovalent cations is larger. Therefore, the same amount of sulfonate groups within the MOF channels attracts and permeates more Mg^2+^ ions than Na^+^, as shown in Fig. 5a. The results further prove that the as-synthesized U-SM(X)s are a continuous membrane with high separation performance compared with that of blank AAO (Figs. 5c and S7a).

We have also tested the ions separation performance for K^+^/Mg^2+^ and Li^+^/Mg^2+^ systems. We found that the separation of K^+^/Mg^2+^ and Li^+^/Mg^2+^ is very different from that of Na^+^/Mg^2+^. Although the membranes exhibit an increasing monovalent cation permeation, the permeation of Mg^2+^ increased even more, which results in the decreased selectivity obviously. We expected to explore the mechanism of this phenomenon by further investigating the permeation of single-component solution (0.1 mol L^−1^; NaCl, KCl, LiCl, and MgCl_2_) during ED. Representative U-SM(25) was selected for single-component ionic transport evaluation due to the highest separation performance in separating Na^+^/Mg^2+^ (Fig. 5d). The permeation of single-component solution precisely depends on the hydrated radii, which follows the order of K^+^>Na^+^ > Li^+^ > Mg^2+^ [11]. The ideal selectivity [13, 39] obtained from the ratio of the permeation of single-component solution (K^+^/Mg^2+ ^= 5091, Na^+^/Mg^2+ ^= 2449, Li^+^/Mg^2+ ^= 776) is much higher than the selectivity of binary solutions (K^+^/Mg^2+ ^= 5.31, Na^+^/Mg^2+ ^= 170, Li^+^/Mg^2+ ^= 1.88). In the binary separation solutions, the permeation of K^+^, Na^+^, and Li^+^ decreased; this result is as expected. However, the permeation of Mg^2+^ increases drastically, especially in K^+^/Mg^2+^ and Li^+^/Mg^2+^, which leads to the significant decrease in selectivity. It is deduced that the ion–ion interaction, ion–water interaction, or wall–ion interaction would affect the ions transfer, which has been confirmed in previous research works [40–42]. However, the specific reason in our case is still unclear and needs more investigation.

## Conclusions

Our results indicate that leaf-like UiO-66-SO_3_H membranes are extremely promising to improve cation permeation and achieve high selectivity. Such ultrathin and defect-free MOF-CPMs contain multidimensional sub-nanometer pores, which are highly suitable for selective transport and separation of ions. Simultaneously, these MOF-CPMs have high monovalent ion permeation (three times of UiO-66 membrane) due to the introduction of the permeation assisting agents (sulfonate) in MOF nanostructures, which could accelerate the cation transport. Consequently, the fabricated MOF-CPMs exhibit excellent Na^+^/Mg^2+^ cation permselectivity (> 140) as well as high monovalent permeation. Thus, the in situ growth of leaf-like UiO-66-SO_3_H membranes has great potential in the cation separation process, which infers importance of introducing sulfonic acid groups in MOF-CPMs.

## Electronic supplementary material

Below is the link to the electronic supplementary material.

Supplementary material 1 (PDF 886 kb)
