# Supplementary material for: Engineering Leaf-Like UiO-66-SO3H Membranes for Selective Transport of Cations
Source: Nanomicro Lett. 2020 Feb 17;12:51. doi: 10.1007/s40820-020-0386-6 (PMC7770750; doi:10.1007/s40820-020-0386-6)
Supplement: Supplementary file 1 — Supplementary material 1 (PDF 886 kb) [file 40820_2020_386_MOESM1_ESM.pdf]

Supporting Information for

Engineering Leaf-like UiO-66-SO_3H Membranes for Selective Transport of Cations

Tingting Xu[1], Muhammad Aamir Shehzad[1], Xin Wang[1], Bin Wu[2], Liang Ge[1,] *, Tongwen Xu[1,] *

[1]CAS Key Laboratory of Soft Matter Chemistry, *i*CHEM (Collaborative Innovation Center of Chemistry for Energy Materials), Department of Applied Chemistry, School of Chemistry and Materials Science, University of Science and Technology of China, Hefei 230026, People's Republic of China

[2]School of Chemistry & Chemical Engineering, Key Laboratory of Environment-Friendly Polymeric Materials of Anhui Province, Anhui University, Hefei 230601, People's Republic of China

*Corresponding authors. E-mail: geliang@ustc.edu.cn (Liang Ge); twxu@ustc.edu.cn (Tongwen Xu)

Supplementary Figures and Tables

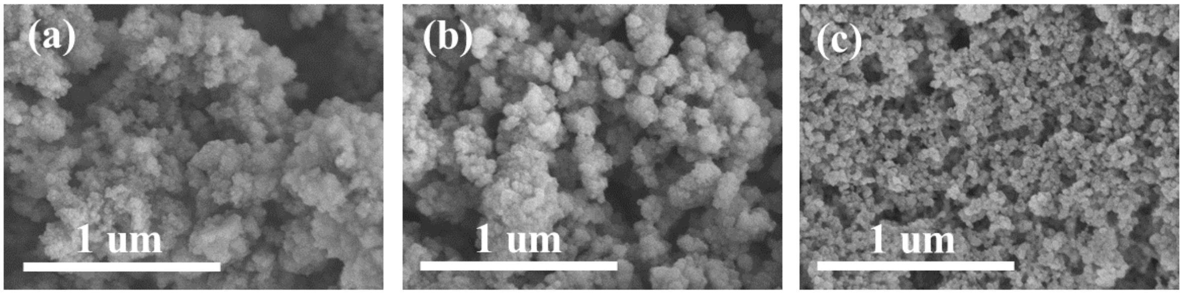

Fig. S1 SEM images of different growth acid environments for UiO-66-SO_3H, **a** without acid, **b** with 8 mL CH_3COOH, **c** with 8 mL HCl

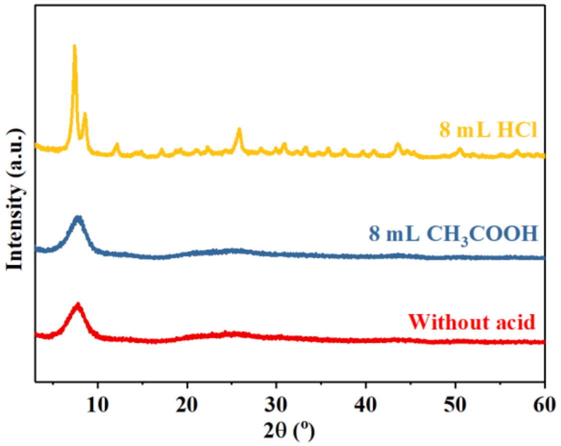

Fig. S2 XRD patterns of different growth acid environments for UiO-66-SO_3H

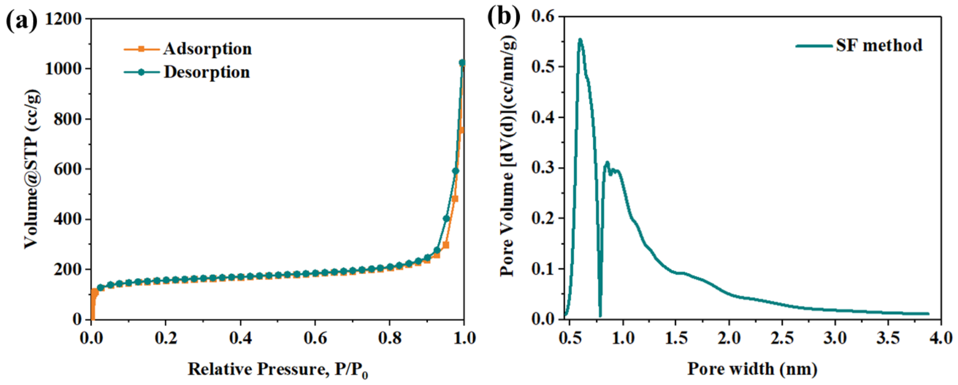

Fig. S3 a N_2 adsorption measurement and **b** pore size distribution of for UiO-66-SO_3H prepared with 8 mL HCl

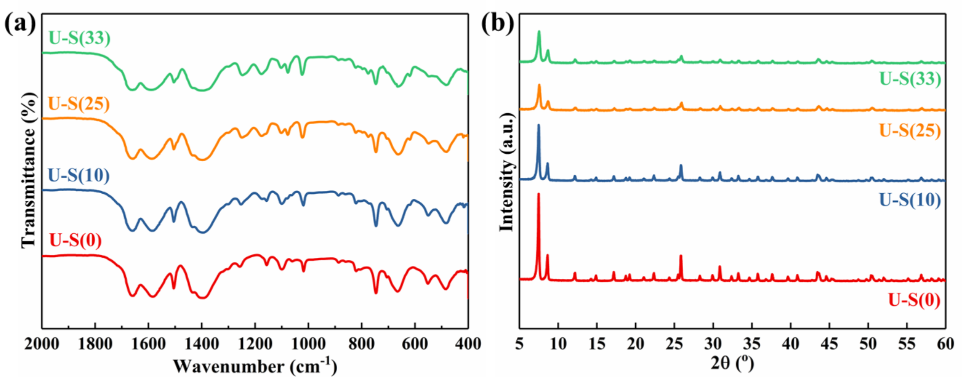

Fig. S4 a FTIR, **b** XRD patterns for 120 h stability test of U-S(X)s

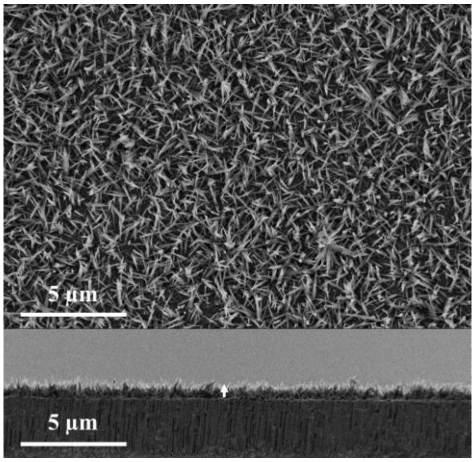

Fig. S5 SEM image of the surface and cross section of U-SM (25) membrane at low magnification. The SEM micrographs confirm good coverage of the AAO surface with UiO-66-SO_3H leaves

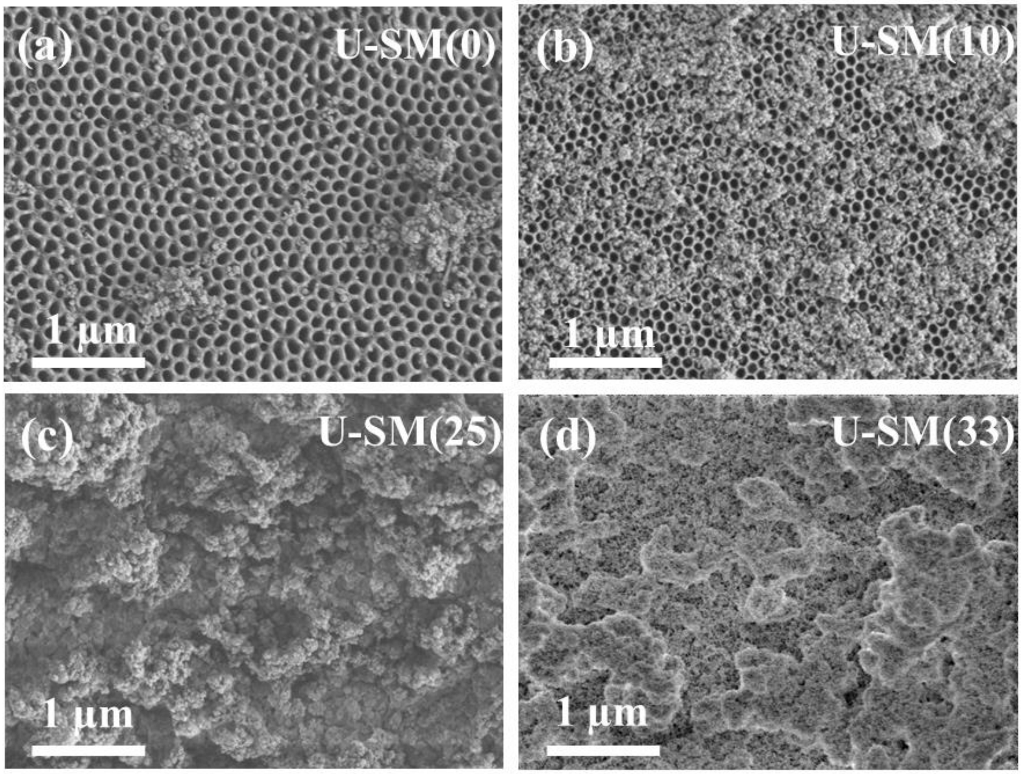

Fig. S6 SEM surface images facing to salt solution side of **a** U-SM(0), **b** U-SM(10), **c** U-SM(25), and **d** U-SM(33)

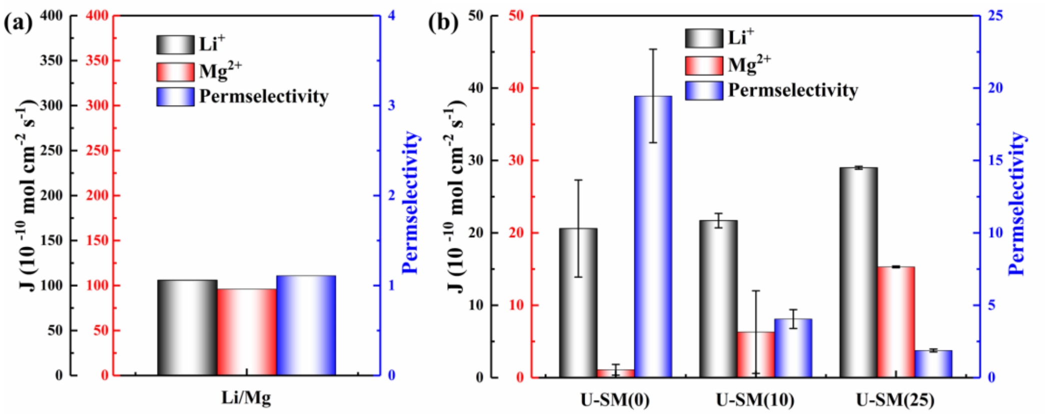

Fig. S7 Li^{+}/Mg^{2+} separation performance of **a** blank AAO, **b** U-SM(X)s

Table S1 BET surface area of U-S(X) nanoparticles

U-S(X)	U-S(0)	U-S(10)	U-S(25)	U-S(33)
**surface area (m^2/g)**	1118	1094	837	806

Table S2 Recipe for UiO-66-SO_3H membranes prepared with different sulfonate content

Membranes	Reaction time (h)	Reaction concentration of 2-$NaSO_3$-H_2BDC (g L^{-1})	Reaction concentration of H_2BDC (g L^{-1})	Reaction concentration of $ZrCl_4$ (g L^{-1})
U-SM(0)	24	0	8.3	11.65
U-SM(10)	24	1.34	7.47	11.65
U-SM(25)	24	3.35	6.23	11.65
U-SM(33)	24	4.49	5.52	11.65
